# Social attention during object engagement: toward a cross-species measure of preferential social orienting

**DOI:** 10.1186/s11689-022-09467-5

**Published:** 2022-12-14

**Authors:** Claire Weichselbaum, Nicole Hendrix, Jordan Albright, Joseph D. Dougherty, Kelly N. Botteron, John N. Constantino, Natasha Marrus

**Affiliations:** 1 Department of Psychiatry, Washington University School of Medicine, 660 S. Euclid Ave, Box 8504, St Louis, MO 63110 USA; 2 Department of Genetics, Washington University School of Medicine, 660 S. Euclid Ave, Box 8232, St Louis, MO 63110 USA; 3 Department of Pediatrics, Marcus Autism Center, Emory University Pediatric Institute, 1920 Briarcliff Rd, Atlanta, GA 30329 USA; 4 Virginia Tech Autism Clinic​ & Center for Autism Research, Virginia Polytechnic Institute and State University, 3110 Prices Fork Rd, Blacksburg, VA 24060 USA; 5 Department of Radiology, Washington University School of Medicine, 660 S. Euclid, 35 Ave, St Louis, MO 63110 USA

**Keywords:** Autism, Social orienting, Social attention, Cross-species, Social motivation

## Abstract

**Background:**

A central challenge in preclinical research investigating the biology of autism spectrum disorder (ASD) is the translation of ASD-related social phenotypes across humans and animal models. Social orienting, an observable, evolutionarily conserved behavior, represents a promising cross-species ASD phenotype given that disrupted social orienting is an early-emerging ASD feature with evidence for predicting familial recurrence. Here, we adapt a competing-stimulus social orienting task from domesticated dogs to naturalistic play behavior in human toddlers and test whether this approach indexes decreased social orienting in ASD.

**Methods:**

Play behavior was coded from the Autism Diagnostic Observation Schedule (ADOS) in two samples of toddlers, each with and without ASD. Sample 1 (*n =* 16) consisted of community-ascertained research participants, while Sample 2 involved a prospective study of infants at a high or low familial liability for ASD (*n =* 67). Coding quantified the child’s looks towards the experimenter and caregiver, a social stimulus, while playing with high-interest toys, a non-social stimulus. A competing-stimulus measure of “Social Attention During Object Engagement” (SADOE) was calculated by dividing the number of social looks by total time spent playing with toys. SADOE was compared based on ASD diagnosis and differing familial liability for ASD.

**Results:**

In both samples, toddlers with ASD exhibited significantly lower SADOE compared to toddlers without ASD, with large effect sizes (Hedges’ *g* ≥ 0.92) driven by a lower frequency of child-initiated spontaneous looks. Among toddlers at high familial likelihood of ASD, toddlers with ASD showed lower SADOE than toddlers without ASD, while SADOE did not differ based on presence or absence of familial ASD risk alone. SADOE correlated negatively with ADOS social affect calibrated severity scores and positively with the Communication and Symbolic Behavior Scales social subscale. In a binary logistic regression model, SADOE alone correctly classified 74.1% of cases, which rose to 85.2% when combined with cognitive development.

**Conclusions:**

This work suggests that a brief behavioral measure pitting a high-interest nonsocial stimulus against the innate draw of social partners can serve as a feasible cross-species measure of social orienting, with implications for genetically informative behavioral phenotyping of social deficits in ASD and other neurodevelopmental disorders.

## Background

Opportunities for engagement with the social world present soon after birth. During neurotypical development, infants preferentially orient toward social stimuli in their environment, showing greater attention to faces and face-like patterns [1, 2] and preference for faces with direct rather than averted gaze [3]. Instances of preferential social orienting, which emerge as more reflexive behaviors during infancy, prompt adult caregivers to respond contingently with facial expressions and sounds that provide the infant with early social communicative experiences [4]. This reflexive infant orienting transitions to volitional gaze toward faces, allowing infants to experience the opportunity to control the contingency between orienting and caregiver response [4]. As an early-emerging social attentional bias, social orienting is theorized to represent an aspect of social motivation, the drive that compels species toward affiliative and cooperative behaviors [5] and is hypothesized to provide a foundation for learning skills contributing to later social communication, such as joint attention [6, 7]. In keeping with this hypothesis, children with autism spectrum disorder (ASD), a neurodevelopmental condition clinically defined by social communication deficits, show characteristic differences in social orienting, including reduced eye contact and response to name as well as impaired joint attention prior to diagnosis [7–11].

A large body of eye-tracking literature supports that in ASD, divergence in the development of social attentional bias from a neurotypical trajectory occurs prior to toddlerhood, when ASD can first be diagnosed. Objective quantification of social orienting using eye-tracking while children view dynamic video clips has found a decline in social orienting to eyes within the first year of life among infants who develop ASD [12]. Similarly, toddlers with ASD spend less time attending to social stimuli like eyes and faces compared to controls [13, 14], and social orienting has been shown to correlate with social competence in children with ASD [15]. Contextual factors influencing social orienting in children with ASD have been clarified by competing-stimulus eye-tracking paradigms, which present social and nonsocial visual stimuli simultaneously and compare the proportion of time spent looking at each stimulus type [16–18]. These studies have observed that although young children with ASD, like controls, generally orient toward people and items in their visual field, their gaze towards social stimuli is more strongly reduced in the presence of social bids for attention (e.g., directed speech and eye contact) and more salient nonsocial items related to intense or restricted interests [16, 18]. These competing-stimulus eye-tracking metrics of social orienting have shown utility as a predictive marker of ASD and ASD severity [17, 19–21].

Naturalistic tasks relying on competing-stimulus approaches have also been informative for demonstrating how simultaneous information in the environment can highlight relative deficits in social orienting for children with versus without ASD. In one experiment, when toddlers were given a tablet displaying video clips, toddlers with ASD were less likely to re-orient towards an experimenter calling their name compared to neurotypically developing or developmentally delayed toddlers [22]. In another experiment, six-month-old infants with high familial liability for ASD made fewer spontaneous looks towards a caregiver than controls when presented with a musical toy [23]. These findings align with observations from competing-stimulus eye-tracking studies demonstrating reduced social orienting in the context of greater attention to nonsocial stimuli among subjects with ASD compared to neurotypically developing controls [18, 24].

Social orienting represents a promising ASD biomarker given it both indexes individual differences relevant to ASD symptoms [19] and is genetically influenced [25–27]; moreover, decreased social orienting in infants may serve as a predictive marker of ASD recurrence in infants with a family history of ASD [7]. Recent evidence supports that both ASD [28] and social orienting [29] are also subject to epigenetic regulation, whereby experiences lead to modifications of the genome that alter gene expression but not the DNA sequence. In infants, profiles of DNA methylation, a common epigenetic modification, have been associated with subsequent ASD diagnoses [30] and ASD polygenic risk scores [31]. Characterization of genetic, epigenetic, and neural correlates of social orienting, including those identifiable prior to ASD diagnosis, may thus elucidate mechanisms contributing to social communication impairment in ASD and the development of affiliative behaviors more generally.

In humans, however, studies of how genetic and epigenetic factors influence neurodevelopment, as well as how early-emerging capacities like social orienting may promote the development of social communication, are constrained by practical and ethical limitations on monitoring and controlling children’s naturalistic environments and probing underlying neural circuitry. Preclinical animal models, which are amenable to testing hypotheses that integrate the impact of genetics and environment on evolutionarily conserved elements of social development, therefore provide a valuable approach for characterizing the biology of ASD. Recent work on chimpanzee and macaque adaptations of the Social Responsiveness Scale, a human measure of quantitative autistic traits, has demonstrated the feasibility of cross-species phenotyping of ASD-relevant social behaviors [32, 33]. Among social behaviors affected in ASD, social orienting is a promising target for cross-species phenotyping given that it is evolutionarily conserved in affiliative species such as chicks, nonhuman primates, and dogs, which like humans, show a preference for faces of conspecifics [34–36] and biological motion [37–39]. Eye-tracking studies in young macaques have also found that attention to social stimuli predicts later social abilities [40], analogous to observed relationships in humans between social orienting and social competence [15], and is diminished by ASD-associated perturbations such as maternal immune activation [41]. Brain regions responsible for processing social stimuli such as biological motion are similar between humans and nonhuman primates [42], supporting the translational relevance of animal models to explore neural underpinnings of social orienting. However, cross-species measures of social orienting are currently limited given the challenge of developing behavioral assays that are comparable between humans and other animals. Factors contributing to this challenge include less elaborated social-cognitive abilities, e.g., in rodents, as well as an evolutionary history of distinct selective pressures within disparate environmental and social contexts for other mammalian species, e.g., non-human primates [43].

The domestic dog, or *canis familiaris*, has emerged as a tractable animal model for ASD [43]. Increasing evidence suggests that the co-evolution of humans and canines entailed in domestication has allowed adaptations in socio-cognitive functioning and selection of social traits, such as high responsiveness and reward sensitivity to human social signals, that have resulted in interspecific social competence between domestic dogs and humans [43, 44]. Because domestic dogs have been encultured in human society, evolutionary changes in canine genetic architecture, as well as epigenetic processes prompted by shared environment, may exhibit convergence with the biology underlying social orienting in humans. Human-directed sociability and sensitivity to human communication exhibit heritable influences in domestic dogs [45, 46], which display a variety of human-analog social abilities during canine-human interactions including sensitivity to human emotional facial expressions [47], initiation of eye contact [48], gaze following [49], and production of referential cues [50]. While several of these behaviors also occur in non-human primates, humans’ evolutionarily closest animal relative, in domestic dogs, they are distinguished by greater sophistication and flexibility [44, 51]. Unlike non-human primates, dogs have been shown to integrate visual and auditory cues to discriminate positive and negative emotions in both conspecifics and humans [52], to infer referents from human gaze [49, 53], and to locate hidden food based on a variety of human social communicative cues [54].

Recent findings have also shown overlapping neurobiological underpinnings for social behavior in humans and human-oriented social behavior in domestic dogs. For example, the amygdala and insula, substrates of affective processing in humans, have been implicated in neural responses to salient human social stimuli in domestic dogs [55]. Like humans, dogs demonstrate increased social responsiveness to humans when exposed to oxytocin, a neuropeptide hormone that enhances affiliative behavior in vertebrates [56]. Increased DNA methylation of the oxytocin receptor gene has been associated with ASD [57] and lower neural responsiveness to social stimuli in humans [29], as well as reduced approach towards unfamiliar humans in dogs [58]. These parallels in phenomenology and mechanism highlight the unique viability of canine behavioral models to measure ASD-relevant traits that may share common genetic, epigenetic, or neural mechanisms with ASD.

Dogs’ coevolutionary history with humans also suggests that naturalistic studies of social behavior in domestic dogs can be conducted based on dogs’ interactions with humans [59], as demonstrated by human-directed sociability tasks [60–63]. In these paradigms, domestic dogs have shown preferential orienting towards humans which can vary according to familiarity with the experimenter [62, 63], the use of human social cues such as pointing [61], and prior actions of a human partner [64]. This profile of social attention to humans corresponds to behavior observed in neurotypical human infants and young children [43, 44, 51], lending additional support to the applicability of these canine tasks to early childhood studies of altered social orienting in neurodevelopmental conditions such as ASD. Consistent with this notion, a recent competing-stimulus index of the degree to which dogs oriented towards a human social partner versus a highly engaging nonsocial distractor was genetically informative, as it associated with structural variants in several genes within the canine ortholog of the Williams-Beuren Syndrome locus. Within this region, duplication in humans leads to neurodevelopmental syndrome featuring autistic-like behavior, whereas deletion is associated with hypersociability, suggesting a dosage-related effect on social function [62]. This genetic finding implies the opportunity to leverage a competing-stimulus approach, which has anchored metrics of social orienting in both human infants (via eye-tracking studies) and canines, as a comparative cross-species metric of social orienting. Such a metric could ultimately provide a tool to identify evolutionarily conserved neural and genetic correlates of this behavioral domain, including those implicated in ASD, as well as disentangle downstream social learning opportunities, and potential associated epigenetic processes, that may contribute to the development of ASD.

In the present study, we developed and tested a competing-stimulus measure, “Social Attention During Object Engagement” (SADOE), which we modeled on the genetically informative competing-stimulus index of social orienting developed in canines [62]. To investigate whether SADOE could distinguish toddlers with and without ASD, we quantified SADOE in two samples, a group of community-ascertained toddler research participants with and without ASD and a prospective study of infant siblings at high and low familial liability for ASD. SADOE was quantified in the context of free play, a naturalistic activity enhancing ecological validity, which is incorporated within the Autism Diagnostic Observation Schedule (ADOS), a semi-structured diagnostic assessment for ASD [65, 66]. To index SADOE, we isolated segments of play in which the child engaged with pre-selected high-interest toys and then coded the frequency of the child's social looks during this period. We hypothesized that, in agreement with previous human and canine studies, the presence of a salient nonsocial object competing with the attentional draw of a social partner would provide sufficient dynamic range to capture individual differences in social orienting and that SADOE would be reduced among toddlers with ASD as well as toddlers with elevated familial ASD liability.

## Methods

### Participants

#### Sample 1 – community-ascertained sample of toddlers with ASD

SADOE coding procedures were initially tested in a subsample of children with (*n =* 8) or without ASD (*n =* 8, see Table 1 for demographics) who were enrolled in a longitudinal research studies on the development of ASD and ASD traits [67, 68]. Comparison participants without ASD included toddler twins from the general population, who were epidemiologically ascertained using the Missouri Family Register, a statewide population-representative twin birth registry [67]. Toddlers with ASD were identified based on either having a community diagnosis or suspected diagnosis of ASD. Research confirmation of a clinical-best-estimate ASD diagnosis was obtained using the ADOS conducted by research-reliable assessors. Participants included in analyses had valid and codable video-recorded ADOS evaluations performed between age 18 months and 3 years.

**Table 1:** Sample characteristics

	**Sample 1: Toddlers with/without ASD**		**Sample 2: Prospective Infant Sibling Study**		
	Non-ASD (*n =* 8)	ASD (*n =* 8)	LL- (*n =* 19)	HL- (*n =* 33)	HL + (*n =* 15)
Age in months	36.3 (1.0)	36.7 (0.5)	24.4 (0.4)	24.8 (1.6)	24.5 (0.6)
Males/Females	5/3	4/4	10/9	13/20	10/5
Race					
Caucasian	6	5	11	24	14
African American	0	3	0	1	0
Biracial	1	0	1	0	0
Other Mixed Race	1	0	1	3	0
Not reported	0	0	7	5	1
Ethnicity (Hispanic yes/no)	0/8	0/8	1/18	2/31	1/14
Mullen ELC	92.8 (28.2)	64.6 (11.4)	105.5 (12.9)	99.8 (13.9)	74.9 (17.8)
ADOS total score	2.0 (1.1)	16.6 (3.6)	2.5 (2.3)	3.0 (2.4)	15.5 (4.8)
ADOS social affect score	1.0 (0.8)	12.0 (2.0)	2.1 (2.3)	2.4 (2.2)	11.9 (4.9)
ADOS CSS social affect score	1.1 (0.4)	7.1 (1.6)	1.7 (1.0)	2.0 (1.2)	5.9 (2.1)
CSBS total score	n/a	n/a	103.5 (12.3)	100.9 (15.0)	74.1 (12.8)
CSBS social composite score	n/a	n/a	48.4 (5.9)	46.6 (6.6)	25.2 (11.8)

Numbers presented as mean (standard deviation).

*ASD* Autism spectrum disorder, *non-ASD, without ASD; ADOS* Autism diagnostic observation schedule, *CSS* Calibrated severity score, *ELC* Early Learning Composite score from the Mullen Scales of Early Learning, *CSBS* Communication and Symbolic Behavior Scales, *LL-* Low familial ASD likelihood and negative for ASD, *HL-* High familial ASD likelihood and negative for ASD, *HL +*  High familial ASD likelihood and positive for ASD

#### Sample 2 – prospective infant sibling study of ASD

ADOS videos from a subsample of toddlers (*n =* 67) enrolled at the Infant Brain Imaging Study (IBIS) at the Washington University in St. Louis site [69] were coded (see Table 1 for demographics). IBIS is a prospective study of infant siblings at low and high familial liability for ASD based on having an older sibling with ASD. Given the established high heritability of ASD, familial liability is likely to reflect the influence of inherited genetic factors for ASD [70]. The family study design of IBIS allowed participants to be classified into three groups at differing likelihood of having an ASD diagnosis, listed here in ascending order of familial ASD liability: 1) the low-likelihood negative group (LL-; *n =* 19), comprised of infant siblings without an ASD diagnosis or a first- or second-degree family history of ASD, 2) the high-likelihood negative group (HL-; *n =* 33), comprised of infant siblings without an ASD diagnosis who had an older sibling with ASD, and 3) the high-likelihood positive group (HL + ; *n =* 15), comprised of infant siblings with an ASD diagnosis and an older sibling with ASD. Selected participants had a valid, codable video recording of the ADOS conducted at the 24-month study visit. A clinical-best-estimate diagnosis of ASD was made by an experienced examiner based on all available information from the 24-month IBIS behavioral battery, including the ADOS and observations during in-person assessments. Testing, video, and interview data were reviewed by a second experienced experimenter to confirm that criteria for an ASD (Autism or Pervasive Developmental Disorder NOS) were met using the DSM-IV-TR checklist at 24 months.

### Measures

#### Autism Diagnostic Observation Schedule (ADOS)

The ADOS is a semi-structured play-based assessment of social interaction, communication, play skills, and restricted interests/repetitive behavior characteristic of ASD [65]. All participants received the Toddler Module, Module 1, or Module 2 of the ADOS or ADOS-2, administered by a certified examiner with a caregiver present in the room. The ADOS social affect calibrated severity score, which accounts for variation in raw scores that may be related to cognition [71, 72], was used for correlational analyses with SADOE. Higher scores indicate greater ASD-related behaviors.

#### Communication and Symbolic Behavior Scales Developmental Profile, Behavioral Sample (CSBS)

The CSBS behavioral sample is a semi-structured assessment collected in 24-month-old IBIS participants. The protocol consists of interactions between the examiner and child that are designed to elicit specific social and communicative behaviors. This measure is appropriate for children with a functional communication age between 6 to 24 months old [73]. Composite scores are calculated for Social, Speech, and Symbolic domains as well as a combined overall score. Composite social scores were used in correlational analyses with SADOE, with lower scores indicating greater impairment in social communicative behaviors.

#### Mullen Scales of Early Learning (MSEL)

The MSEL is a developmental assessment battery that provides an index of general cognitive development in young children ages birth to 68 months old [74]. Its five subscales target skills in the areas of receptive language, expressive language, visual reception, fine motor, and gross motor. The Early Learning Composite score (ELC) reported here is the sum of four subscales, providing an overall developmental standard score. Lower scores indicate less advanced cognitive development.

### Behavioral coding of social attention during object engagement (SADOE)

The behaviors selected for coding as part of the SADOE paradigm were based on an existing canine behavioral measure of human-directed social attentional bias, an aspect of increased canine sociability toward humans associated with domestication [62]. In this task-based measure, canines were presented with an opportunity to extract a sausage, a highly attractive food item, from a solvable puzzle box. Canines were allowed up to two minutes to attempt to open the box and retrieve the item while in the presence of a familiar human experimenter. To evaluate social attentional bias, the duration of the canines’ gaze toward the human experimenter and the puzzle box were coded. Using this task, domesticated dogs, who have been observed to engage in more prolonged contact with human caretakers than human-socialized wolves [75], were found to spend a greater proportion of time looking at the experimenter versus the box in comparison to wolves. We hypothesized that toddlers with ASD (or elevated familial ASD liability) who participated in an analogous play-based paradigm would display a lower proportion of time engaged in social looking than children without ASD, given characteristic symptoms involving reduced social orienting in ASD.

To translate the canine competing-stimulus paradigm to index naturalistic social attentional bias in toddlers, we coded portions of free play in the ADOS during which time the child was in the presence of their caregiver and an experimenter while interacting with a focal toy. The first three minutes of coded free play occurred at the start of the ADOS and entailed child-directed toy exploration in the absence of adult prompting. This was followed by integration of experimenter and caregiver bids toward the child while the child continued toy exploration. This sample thus allowed observation of the child’s behavior in the context of both self-directed behavior and social prompting, enhancing generalizability across a range of real-world contexts.

Three toys from the standard ADOS materials, a musical pop-up toy, musical piano toy, and animal sounds toy, were selected a priori as focal toys, since, like the puzzle box in the canine task, key sound and visual features of the toys were contingent on the subject’s actions. Further, based on the team’s extensive prior experience administering the ADOS, these toys were broadly appealing and sustained the attention of children with and without ASD. Coded instances of engagement with a focal toy (i.e., object engagement) were defined as at least 10 s of continuous interaction with the toy, beginning with physical contact and ending when the child moved away or engaged with a different object. During these periods, the child’s target of visual attention was coded as the focal toy (toy looking), the examiner or the caregiver (social looking), or none of these (other looking). Social looks were further categorized by their degree of spontaneity, here intended to reflect the initiative required from the child. Spontaneity was categorized based on the presence of potential prompting events for a social look in the preceding three seconds. Prompting events entailed actions by the examiner or caregiver such as touching the child or calling the child’s name (name/touch code), speech or movement directed toward the child (child-directed code), and speech or movement that was not specifically directed at the child (non-child-directed code). If none of these prompting events occurred in the three seconds before the look, the social look was considered spontaneous. In SADOE coding for Sample 1, social looks were coded as spontaneous or not spontaneous, as this sample provided initial testing for the SADOE measure. In Sample 2, the larger sample, non-spontaneous social looks were further specified as name/touch, child-directed, or non-child-directed.

The frequency and duration of each look type was calculated using the BORIS software package [76], which allows the user to flag a customized set of behaviors within a video. Participants were excluded if they interacted with the designated focal toys for less than one minute total or if their eyes were not consistently visible in the video recording. Raters were blinded to diagnosis and demonstrated high inter-rater reliability, established by co-coding an independent sample of ten videos (ICC = 0.93 for social looks, ICC > 0.84 across all look types; see Table 2). Indices of SADOE were calculated by dividing the number of each social look type by the total duration of focal toy engagement.

**Table 2:** Inter-rater reliability of video coding

Behavioral Code	ICC Value
Time Engaged with Focal Toy	.92
Toy Looks	.84
Social Looks—Response to Touch or Name	.98
Social Looks—Response to Child-Directed Speech or Movement	.92
Social Looks—Response to Non-Child-Directed Speech or Movement	.99
Social Looks – Spontaneous	.96
All Social Looks	.93

Single measures consistency intraclass correlation coefficients (ICC) were calculated with a two-way random effects model, from an independent sample of ten videos coded by two raters blind to diagnosis

### Statistics

Indices obtained as part of coding SADOE were compared across groups of toddlers with and without ASD using independent samples *t*-tests performed in SPSS, with corrected *p-*values applied when group variances were significantly different based on Levene’s test for equality of variances. Effect sizes were calculated using Hedges’ *g* to account for Sample 1’s *n* < 20 participants. Primary analyses involved measurement of SADOE as the frequency of social looks during engagement with focal toys, while secondary analyses involved subcategories of social looks. Power calculations conducted using G-power indicated that at alpha = 0.05 with 80% power, both samples 1 and 2 were powered to detect large effect sizes (for Sample 1, effect size ≥ 1.5; for Sample 2, effect size ≥ 0.9). Spearman’s correlation was used to evaluate a hypothesized inverse relationship between SADOE and ordinal levels of ASD-related behaviors from ADOS social affect calibrated severity scores, while Pearson’s correlation was used to evaluate a hypothesized positive relationship between SADOE and continuous social scores on the CSBS. Binary logistic regression using pooled samples to maximize power tested whether 24-month SADOE was a concurrent predictor of ASD diagnosis. For all regression models, Hosmer-Lemershow tests were not significant, consistent with appropriate model fit. Variance was reported based on Nagelkerke’s pseudo-R^2^.

## Results

### Sample 1: comparison of SADOE in toddlers with and without ASD

**Fig. 1:**
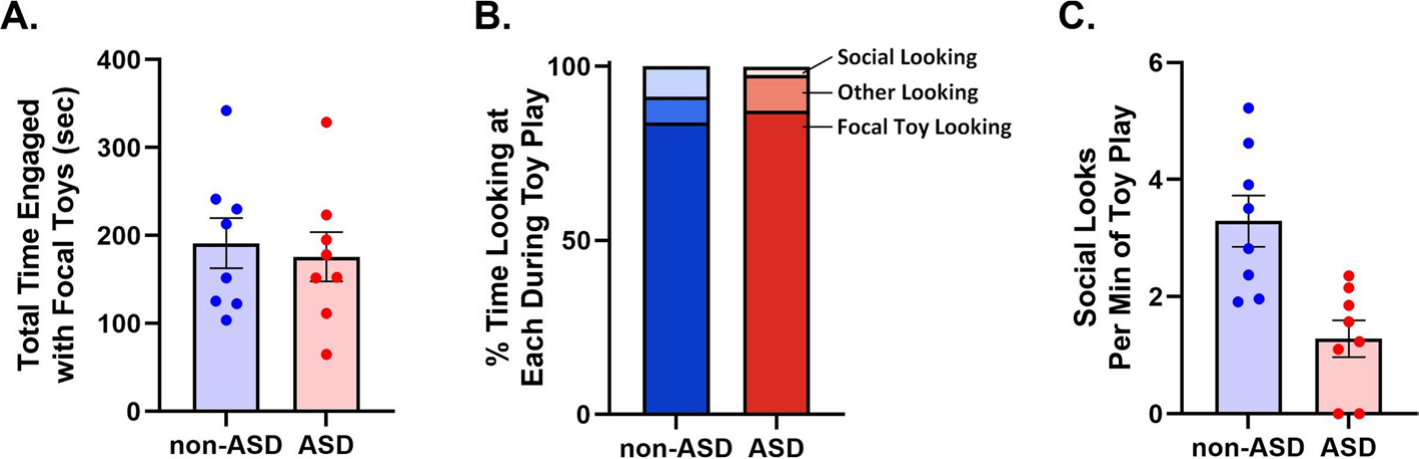
Social Attention During Object Engagement (SADOE) is lower among children with versus without ASD. (A) Total time engaged with focal toys was not significantly different between groups (bars show mean ± 1 SE). (B) Proportions of time each group spent looking at social and nonsocial targets during engagement with the focal toys. (C) Non-ASD children made significantly more social looks per minute during engagement with the focal toys than children with ASD (p = .023; bars show mean ± 1 SE). non-ASD, without ASD; ASD, autism spectrum disorder; SE, standard error. *N =* 8 each for non-ASD and ASD groups

Children with and without ASD were highly engaged with the focal toys, showing similar average durations of time in toy play (non-ASD = 3.5 min, ASD = 3.2 min; *t*(14) = 0.39, *p* = 0.70; Fig. 1A). While playing, children in both groups spent most of the time looking at the toy (non-ASD = 84%, ASD = 87%; *t*(14) = -0.95, *p* = 0.65; Fig. 1B) versus the experimenter, caregiver, or other aspects of the environment (Fig. 1B). However, the non-ASD group exhibited a significantly higher frequency of social looks than the ASD group (non-ASD = 3.3 looks per min, ASD = 1.3 looks per min; *t*(14) = 3.72, *p* = 0.002; Fig. 1C), indicating a reduced tendency for SADOE among toddlers with ASD. The effect size for this difference was large: Hedges’ *g* = 1.76, with 95% confidence intervals (CI) of 0.61 and 2.87. Similarly, the percentage of time spent on social looks differed between the groups, with the ASD-diagnosed toddlers spending less of their time looking at the experimenter and caregiver (non-ASD = 9% ASD = 2%; *t*(9) = 3.50, *p* = 0.006; Hedges’ *g* = 1.66; Fig. 1B).

In terms of spontaneous looks based on the child’s self-directed initiative, toddlers without ASD made four times as many spontaneous social looks per minute than toddlers with ASD [non-ASD = 2.1 looks per min, ASD = 0.5 looks per min; *t*(8) = 2.77, *p* = 0.023; Hedges’ *g* = 1.31]. In contrast, the groups did not significantly differ in terms of social looks that were not spontaneous (non-ASD = 1.2 looks per min, ASD = 0.58 looks per min; *t*(14) = 1.01, *p* = 0.33). Overall, these results illustrate that toddlers with ASD are less attentive to social partners during object engagement than children without ASD.

### Sample 2: comparison of SADOE in toddlers with differing familial ASD liability

Given the differences between toddlers with and without ASD in Sample 1, we next tested for differences among children categorized by level of family liability for ASD in the larger IBIS sample. Here, the LL- group had the lowest familial risk for ASD, followed by the HL- group, and lastly the HL + group, with high familial risk and an ASD diagnosis. By stratifying according to group-level familial ASD risk, we were able to investigate whether differences in SADOE were primarily associated with ASD diagnosis or ASD familial liability.

**Fig. 2:**
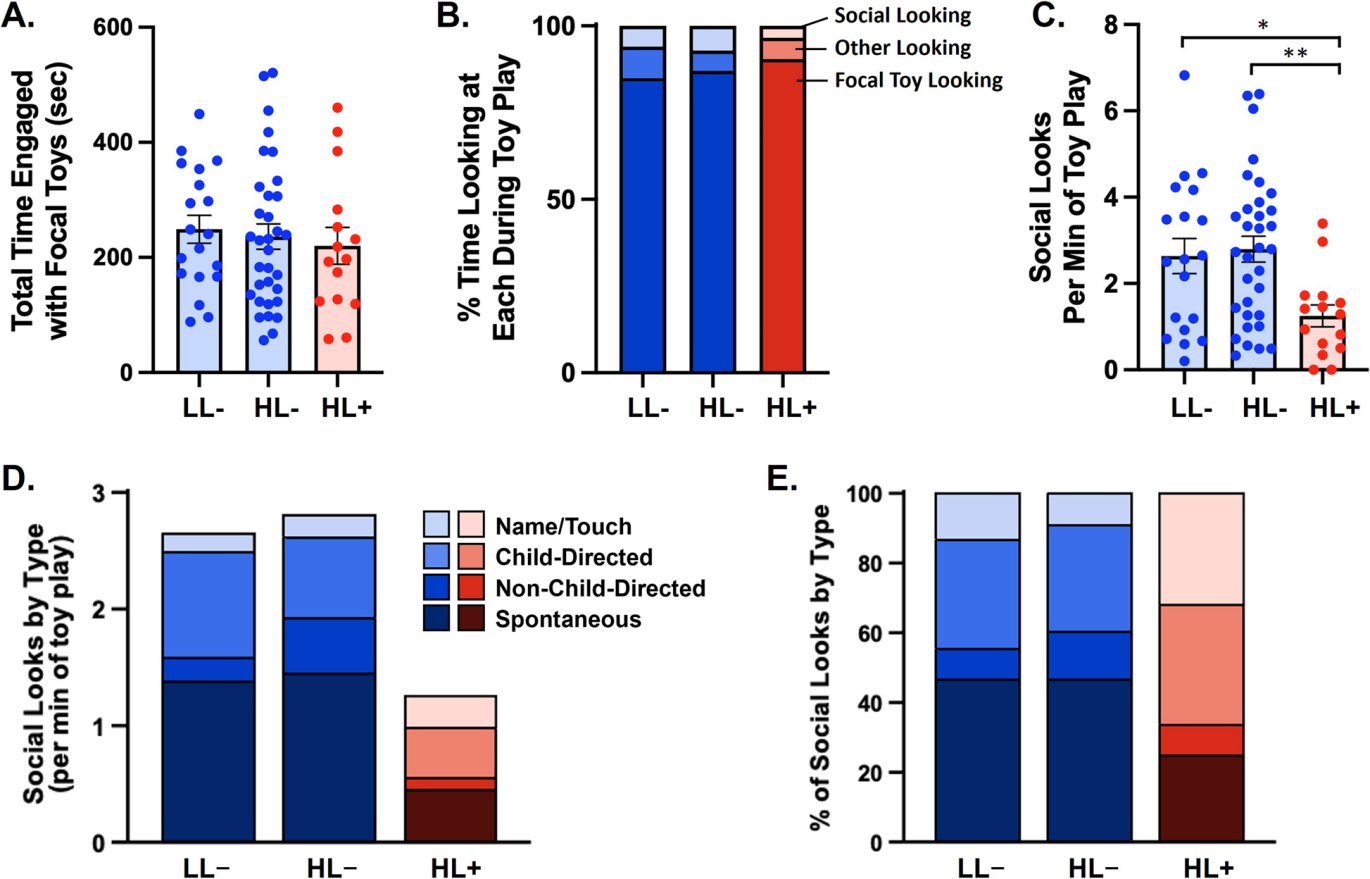
Social Attention During Object Engagement (SADOE) is greater among both high-likelihood (HL) and low-likelihood (LL) toddlers without ASD compared to toddlers with ASD. (A) Total time engaged with focal toys was not significantly different between groups (bars show mean ± 1 SE). B) Proportions of time each group spent looking at social and nonsocial targets during engagement with the focal toys. (C) HL- and LL- groups made more social looks per minute during play with the focal toys compared to the HL + group with ASD (LL- vs. HL + , *p* = .007; HL- vs. HL + , *p* < .001), with no significant difference in LL- versus HL- groups (*p* = .75; bars show mean ± 1 SE). Single asterisk indicates *p* < .01. Double asterisk indicates *p* < .001. (D) Number of social looks per minute by type for each group. Among social look types, the HL + group was lower than both the LL- and HL- groups specifically for spontaneous social looks (*p’s* ≤ .012). (E) Social look types as in (D) but expressed as a percentage of the total number of social looks. The HL + group displayed a lower percentage of spontaneous looks than the LL- and HL- groups (*p’s* ≤ .02). LL-, low-likelihood of ASD without a diagnosis (*N =* 19); HL-, high-likelihood of ASD without a diagnosis (*N =* 33); HL + , high-likelihood of ASD with a diagnosis (*N =* 15). ASD, autism spectrum disorder; SE, standard error

Like Sample 1, all groups demonstrated similar levels of engagement with focal toys (LL- = 4.2 min, HL- = 3.9 min, HL +  = 3.7 min; *p’s* > 0.47; Fig. 2A) and percentage of time looking at focal toys while engaged in play (LL- = 85%, HL- = 87%, HL +  = 90%; *p’s* ≥ 0.17; Fig. 2B). However, the HL + group with ASD showed the lowest frequency of social looks (Fig. 2C), similar to the ASD group in Sample 1. While the HL + group exhibited 1.2 social looks per minute of toy play, both the HL- group and the LL- group showed significantly more social looking, at 2.8 looks/min [*t*(44) = 3.25, *p* < 0.001] and 2.6 looks/min [*t*(29) = 2.90, *p* = 0.007], respectively. Effect sizes were large for both group differences, with confidence intervals that overlapped corresponding findings in Sample 1: LL- vs. HL + , Hedges’ *g* = 0.92 (95% CI: 0.21, 1.61) and HL- vs. HL + , Hedges’ *g* = 1.00 (95% CI: 0.36, 1.62). The HL + group also spent a lower percentage of time than the LL- and HL- groups on social looking while playing with the focal toys: (LL- = 6% time on social looks, HL- = 7% time on social looks, HL +  = 3% time on social looks). This comprised a significant difference between the HL + and HL- group [*t*(43) = 3.42, *p* = 0.001; Hedges’ *g* = 0.87] and a trend-level difference between the HL + and LL- group [*t*(29) = 1.67, *p* = 0.11; Hedges’ *g* = 0.56]. In contrast, neither the frequency of social looks nor the percentage of social looking time differed between the LL- and HL- groups (*p*’s > 0.75).

As found in Sample 1, the HL + group made significantly fewer spontaneous social looks per minute of toy play (Fig. 2D): 0.5 looks/min for the HL + group, with 1.4 looks/min for both the HL- [*t*(44) = 3.62, *p* < 0.001] and LL- groups [*t*(32) = 2.65, *p* = 0.012; Hedges’ *g* = 0.89 for both comparisons]. The LL- and HL- groups did not differ in spontaneous looks [*t*(32) = -0.2, *p* = 0.84]. Among subtypes of toddler social looking that were prompted by distinct caregiver or examiner behaviors (Fig. 2D, 2E), social looks following child-directed examiner/caregiver behaviors (with separate codes for “name/touch” and “child-directed” for other speech or movement) were significantly lower for the HL + versus the LL- group only [*t*(28) = 2.35, *p* = 0.026; Hedges’ *g* = 0.74]. Social looks following examiner/caregiver speech or movement not directed towards the child (coded as “non-child-directed”) were lower in the HL + versus HL- group only [*t*(41) = 3.44, *p* = 0.001; Hedges’ *g* = 0.75].

When considering the subtype of social looking as a percentage of all social looks (Fig. 2E), the HL + group also showed a lower percentage of spontaneous social looks compared to both LL- and HL- groups: LL-, [*t*(30) = 2.46, *p* = 0.02, Hedges’ *g* = 0.86]; HL-, [*t*(44) = 2.42, *p* = 0.02, Hedges’ *g* = 0.78]. No significant differences were found between the HL + group and LL- or HL- groups, or between LL- and HL- groups, for other subtypes of social looking. Collectively, these findings support the specificity of spontaneous social looks for differentiating toddlers with and without ASD, corroborating results from Sample 1, and indicate that diminished SADOE in toddlers is characteristic of ASD rather than familial liability for ASD.

### Correlation of SADOE with measures of ASD-relevant social behaviors

**Fig.3:**
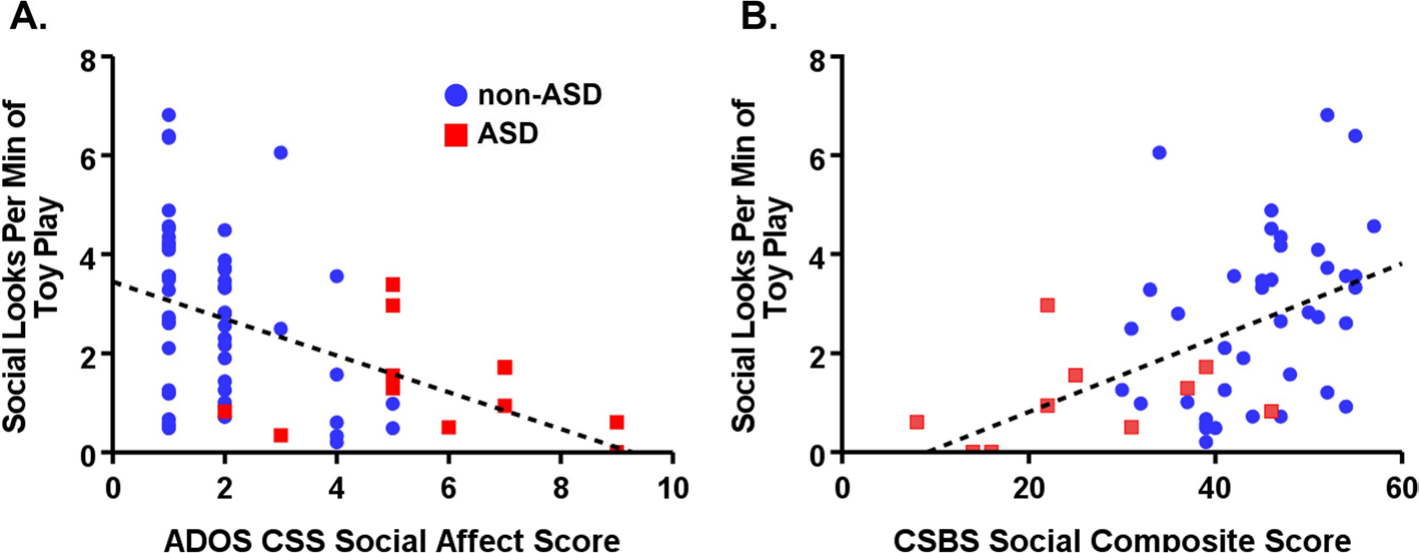
Social Attention During Object Engagement (SADOE) correlates with other measures of social behavior. (A) SADOE was negatively correlated with the ADOS CSS score (*ρ* = -.50, *p* < .001). (B) SADOE was positively correlated with CSBS social score (*r* = .49, *p* = .001). ASD, autism spectrum disorder; non-ASD, without ASD; ADOS, Autism Diagnostic Observation Schedule; CSS, calibrated severity score; CSBS, Communication and Symbolic Behavior Scales

We next investigated whether SADOE is related to existing social behavioral indices commonly used in assessment of children with ASD. SADOE was negatively correlated with ASD-related behaviors on the ADOS calibrated social affect severity score obtained from the same testing sessions across Samples 1 and 2, *ρ* = -0.50, *p* < 0.001 (Fig. 3A). SADOE was also positively correlated with the social composite score of the Communication and Symbolic Behavior Scales (CSBS), a measure of social skills available in Sample 2, *r* = 0.49, *p* < 0.001 (Fig. 3B). These complementary results suggest that SADOE is related but not identical to existing ASD-relevant measures of social behavior.

### SADOE and concurrent prediction of ASD diagnosis

Finally, binary logistic regression based on pooled data across both samples was performed to investigate the diagnostic predictive power of SADOE alone and in combination with the ELC, to assess contributions of cognitive development (Table 3). With SADOE as the only predictor, the model was significant (χ^2^(1) = 18.10, *p* < 0.001) and accounted for 29.4% of the variance in diagnosis while correctly classifying 74.1% of cases as ASD or non-ASD.

**Table 3:** Binary logistic regression for predictors of diagnostic status

	Predictor	B	SE	Wald	*p*	Nagelkerke’s R^2^	% Correctly Classified
Model 1	SADOE	-0.87	0.24	11.85	< .001	0.294	74.1%
Model 2	SADOE	-0.92	0.34	7.21	.007	0.625	85.2%
	ECL	-0.11	0.029	14.83	< .001		

SADOE consists of all social looks per minute of engagement with focal toys

*SADOE* Social attention during object engagement, *ELC* Early Learning Composite score of the Mullen Scales of Early Learning

When the ELC was added to the model, it remained significant (χ^2^(2) = 48.12, *p* < 0.001) and accounted for 65.7% of the variance in diagnosis, with 85.2% of cases being correctly classified by diagnostic status. Both SADOE and ELC were significant contributors to the model (Table 3), suggesting that SADOE provides useful diagnostic information independent of cognitive function for this sample.

## Discussion

Reduced social orienting is a hallmark of ASD, and here we present the first human translation of a straightforward, cross-species method to quantify this phenomenon via a competing-stimulus approach in toddlers. By pitting a highly attractive nonsocial stimulus against the draw of social engagement during unstructured play, this assay enables sensitive quantification of naturalistic social orienting among toddlers with and without ASD. Consistent with a metric representing a core ASD feature, SADOE accounted for a substantial proportion of the variance in diagnosis, and the association between SADOE and diagnostic status remained significant when accounting for the impact of cognitive development. With an average of less than five minutes of play coded per child, this brief assay may be implemented without significant additional burden to families, clinicians, or researchers using the ADOS, a well-established diagnostic tool for ASD.

As predicted, children with ASD did not disengage their attention from the focal toys to look towards their caregiver and the experimenter as often as children without ASD. The effect size for this group difference was large in both samples, consistent with clinical impairment of social orienting in ASD. When toddlers with ASD did make social looks, they were also less likely to be spontaneous and more likely to be elicited by an adult’s behavior. SADOE also correlated in the expected direction with distinct measures of the level of ASD-related behaviors and social communication, in line with prior work showing strong interrelations between social characteristics contributing to ASD traits [27]. Nevertheless, its moderate correlation with ADOS social affect calibrated severity scores and CSBS social composite scores indicates that the SADOE metric captures uniquely informative social variation associated with ASD.

Contrary to our hypothesis regarding SADOE relative to familial liability for ASD, diminished SADOE in toddlers, whether considering all social looks or spontaneous social looks, appears to represent a marker of ASD itself rather than familial liability for ASD. SADOE scores did not differ between LL- and HL- toddlers and score distributions were highly overlapping. Given that decreased social orienting can arise before ASD diagnosis [12], future studies should investigate whether SADOE may stratify familial liability for subsequent ASD earlier in development and predict likelihood of ASD. Such developmentally sensitive differences have been postulated for measures of behaviors which, like social orienting, are heritable [25–27] and hypothesized to contribute to ASD’s ontogeny [77].

The present study provides compelling evidence that children with ASD display significantly less social orienting than their non-ASD peers in the context of a brief, naturalistic engagement with a high-interest nonsocial stimulus. Whether this reflects heightened object attention or diminished social attention remains uncertain; however, there was not a significant difference in the length of time that ASD versus non-ASD children spent engaged with the focal toys, nor in the time they spent looking at the toys during those engagements, suggesting that differences in social looks were not driven by excessive interest in the object. This similarity in attention to the focal toy, along with the strong contribution of spontaneous, self-initiated looks to ASD-associated differences in SADOE, supports the idea that SADOE may be more strongly influenced by social motivation to engage with others rather than object attraction. Of note, a previous study quantified social looking across the full ADOS administration and also found discrepancies in social attention between ASD and non-ASD children [78], consistent with the interpretation that our findings reflect impaired social motivation in ASD rather than simply greater object attraction.

Other measures such as the Early Social Communication Scale (ESCS) [79] present children with novel toys as a means to elicit joint attention and have similarly revealed that children with ASD are less likely to orient towards social partners in that context compared to neurotypically developing peers [80]. Comparable results have been demonstrated in terms of joint attention during naturalistic play [81]. While SADOE focuses on the more basic construct of social orienting, a critical precursor to joint attention, this literature aligns with our finding that children with ASD show reduced social looking during toy play and implies that impaired joint attention may be one downstream consequence of this failure to appropriately attend to social stimuli. Unlike the ESCS and other existing measures, SADOE has the practical advantage of being readily obtainable from video recordings of the widely used ADOS assessment and potentially other observational contexts, regardless of the child’s age or verbal ability. Follow-up studies could also evaluate clinical validity and reliability for a brief prospective SADOE assessment that could augment early ASD screening.

SADOE was intentionally designed to facilitate cross-species behavioral phenotyping by adapting a competing-stimulus paradigm previously established in canines [62] for use in human toddlers to measure an evolutionarily conserved aspect of social behavior implicated in ASD. Observations of reduced SADOE in toddlers with ASD are congruent, as hypothesized, with reduced task-based social orienting in wolves versus domesticated dogs, which unlike wolves, have an extensive co-evolutionary history with humans. This correspondence suggests that competing-stimulus approaches to social orienting designed to be comparable across species (e.g., by accounting for variations in cognitive abilities or socialization with humans versus conspecifics) could be informative in other animal models, such as rodents, with their flexibility to probe molecular genetic mechanisms, and non-human primates, with their high degree of genetic relatedness to humans. Additionally, the present finding, in the context of the association of canine social orienting with a conserved gene linked to human social behavior [62], as well as the known heritability of ASD [70] and human social orienting [25–27], supports strong translational opportunities in canine models. For example, application of SADOE in domestic dogs, which experience an early sensitive period for human socialization [82], could be used to investigate relationships between human exposure, social orienting, and DNA methylation profiles, including in the previously implicated oxytocin receptor gene (29, 57, 58). Such work could generate hypotheses about genes and epigenetic mechanisms playing a role in human social orienting and ASD, while elucidating biological pathways that inform targets for intervention. The feasibility of the competing-stimulus approach in human toddlers further underscores the versatility of canines as a human-analog model, considering the importance of toddlerhood for clarifying the early identification, ontogeny, and subsequent course of ASD symptomology.

Although the retrospective use of existing ADOS videos allowed us to capitalize on existing datasets, it imposed some limitations. While the semi-structured nature of the ADOS assessment moderated the range of possible responses from the experimenter and caregiver, there was still heterogeneity in the behavior of these adults, which could upwardly bias the total number of social looks by increasing children’s opportunity for non-spontaneous social looking. Relatedly, although the toy set was standardized, variables such as the precise placement of the toys and their location relative to social partners were not standardized across subjects. Subjects’ prior exposure to the high-interest toys was also not assessed, potentially adding the confound of familiarity versus novelty for quantifying social orienting relative to engagement with the focal toy. However, the difference in SADOE between ASD and non-ASD subjects was consistent enough to be observed to a similar degree in two independent samples, with differences driven by spontaneous social looks in both cases. Future directions include studies to validate this measure for clinical practice, as well as evaluating how more controlled settings might impact SADOE, as optimizing the balance of ecological validity with experimental control will be most informative for capturing variation in SADOE that differentiates children with and without ASD.

### Conclusions

Our findings suggest that a brief behavioral measure pitting a high-interest object against the innate draw of social engagement can serve as a rapid, feasible measure of social orienting in young children. By developing a human measure that is applicable to other species, this work opens the door to powerful approaches available in model organisms to explore underlying genetic and neurobiological influences on social orienting and how these factors may contribute to mechanisms of ASD and other neurodevelopmental disorders.

## Data Availability

The datasets analyzed during the current study are available from the corresponding author on reasonable request.

## Abbreviations

ADOS: Autism Diagnostic Observation Schedule
ASD: Autism Spectrum Disorder
CSBS: Communication and Symbolic Behavior Scales Developmental Profile, Behavior Sample
ESCS: Early Social Communication Scale
ELC: Early Learning Composite score of the Mullen Scales of Early Learning
HL-: High-Likelihood Negative Group (infant siblings without an ASD diagnosis and an older sibling with ASD)
HL+: High-Likelihood Positive Group (infant siblings with an ASD diagnosis and an older sibling with ASD)
LL-: Low-Likelihood Negative Group (infant siblings without an ASD diagnosis or a first- or second-degree family history of ASD)
MSEL: Mullen Scales of Early Learning
SADOE: Social Attention During Object Engagement
